# Transforming Residual Microbial Biomass into High-Value Bicomposite Material for Reactive-Dye Removal: Insights from Batch Investigations to Fluidized-Bed Reactor Applications

**DOI:** 10.3390/ma19163534

**Published:** 2026-08-20

**Authors:** Daniela Suteu, Alexandra Cristina Blaga, Lacramioara Rusu, Adrian Catalin Puitel, Ramona-Elena Tataru-Farmus

**Affiliations:** 1 Faculty of Chemical Engineering and Environmental Protection “Cristofor Simionescu”, “Gheorghe Asachi” Technical University of Iasi, Blvd. Prof. Dimitrie Mangeron, no. 73, 700050 Iasi, Romania; danasuteu67@yahoo.com (D.S.); adrian-catalin.puitel@academic.tuiasi.ro (A.C.P.); ramona-elena.tataru-farmus@academic.tuiasi.ro (R.-E.T.-F.); 2 Faculty of Engineering, “Vasile Alecsandri” University of Bacau, 157 Calea Mărăşeşti, 600115 Bacau, Romania

**Keywords:** anionic dye Orange 16, biosorbtion, fluidized-bed column, microbial residual biomass, *Saccharomyces pastorianus*

## Abstract

The development of sustainable adsorbents from industrial biowaste has become an important strategy for reducing the environmental impact of both solid waste generation and wastewater pollution. In this study, residual *Saccharomyces pastorianus* biomass recovered from the brewing industry was immobilized in a polymeric matrix and evaluated as a bio-composite for the removal of reactive dyes from aqueous media. Orange 16 was selected as the target molecule. Batch biosorption experiments were conducted to identify the optimum operating conditions and to determine the adsorption capacity through Langmuir isotherm analysis. The performance of the biosorbent was subsequently validated under continuous-flow conditions in a fluidized-bed reactor, where the effects of flow rate on column (3.8 and 8.5 mL/min) efficiency were investigated. Experimental breakthrough curves were analyzed using the Clark, Yan, Bohart–Adams, and Yoon–Nelson models, which adequately described the dynamic biosorption process, particularly at the lower flow rate. The best agreement was obtained at a flow rate of 3.8 mL/min, an initial dye concentration of 87 mg/L, and a biosorbent mass of 19.5 g. The developed bio-composite material exhibited efficient dye removal and stable operation, demonstrating that residual brewing biomass can be successfully transformed into a low-cost and sustainable biosorbent suitable for continuous treatment of reactive-dye-containing wastewaters.

## 1. Introduction

The growing pressure on freshwater resources, driven by rapid socio-economic development and climate change, has accelerated the transition toward sustainable water management. Within the circular economy, wastewater is increasingly regarded not as waste but as a secondary resource, and the valorization of industrial by-products into efficient treatment materials has become an attractive strategy for improving resource efficiency while supporting sustainable water reuse and recycling [1]. By simultaneously addressing wastewater decontamination and the valorization of residual microbial biomass, the present work exemplifies a circular approach to environmental sustainability.

Water pollution remains a critical threat to environmental sustainability and human health, with industrial activities a major contributor through practices that are not always adherent to environmental standards [2]. Industrial effluents carry a broad range of organic pollutants: pharmaceuticals, pesticides, personal-care compounds, and dyes, alongside inorganic contaminants such as heavy metals and engineered nanomaterials, many of which are grouped as emerging contaminants owing to their persistence and their effects even at low concentrations [3]. Conventional treatments, including coagulation–flocculation, precipitation, complexation, ion exchange, and electrochemical techniques, are widely applied [4], yet their performance is frequently constrained by limited efficiency, high cost, operational complexity, and the generation of secondary waste requiring further disposal [2,5]. These constraints have motivated the search for low-cost, sustainable alternatives.

Biosorption has emerged as one such alternative: an effective, environmentally friendly technique for removing toxic organic and inorganic contaminants from wastewater, with demonstrated relevance for mitigating industrial pollution and advancing sustainable water management [6]. It has been applied successfully to diverse effluents, including landfill leachates, municipal and industrial wastewaters, and refinery discharges. Mechanistically, biosorption is a physicochemical, metabolically independent process in which contaminants are passively bound to biological materials such as inactive microorganisms, seaweeds, plant biomass, and agricultural residues [7]. Depending on the sorbate–biosorbent interaction, uptake proceeds through physisorption (weak physical forces between solute molecules and surface binding sites) or chemisorption (formation of chemical bonds with the biosorbent’s functional groups), with absorption, ion exchange, and passive uptake potentially contributing to overall removal [8,9]. The approach is cost-effective, exploits abundant waste biomass, generates minimal secondary pollution, handles large wastewater volumes, and requires no complex equipment; by valorizing biological and agricultural residues, it simultaneously advances waste minimization and resource recovery [7,10,11].

Biosorption processes are typically conducted in two main operational modes: batch (static) and continuous (dynamic). Batch systems are widely studied in the literature and are useful for characterizing the sorbent, understanding adsorption mechanisms, and evaluating factors influencing sorption efficiency [12]. However, they are limited in their applicability for large-scale treatment. In contrast, dynamic biosorption involves the continuous flow of contaminated solution through a column packed with the biosorbent, simulating real industrial conditions. Continuous systems, such as fixed-bed columns, are more relevant for practical applications, enabling long-term operation and efficient removal of pollutants from continuous wastewater flow. Despite the abundance of research on batch systems, reports on the use of biosorbents in dynamic mode remain limited, highlighting the need for further studies to evaluate their performance under continuous processing conditions [13].

In a continuous-flow system, biosorption is conducted in continuous mode, with wastewater streams flowing through a column filled with biological material, allowing pollutants to be retained by the biosorbent [14,15,16]. The efficiency of this process is influenced by multiple parameters, such as the nature of the biosorbent, the flow rate, and the pollutant concentration and composition, as well as the contact time between the contaminants and the biosorbent [15,16,17,18,19,20]. Dynamic biosorption is particularly suited for continuous operation under realistic industrial conditions, allowing sustainable treatment of wastewater while maintaining controlled interaction between the biosorbent and contaminants. To avoid channeling or early saturation of the biosorbent, which may gradually reduce its performance, the system must be carefully monitored and properly maintained [21,22]. Several types of equipment can be employed for continuous-flow experiments [21,23]. Packed-bed systems are the most commonly used, with a column filled with biosorbent through which contaminated water flows at a controlled rate. Despite their widespread use in biosorption, packed-bed reactors have several limitations, including biosorbent saturation, which impairs pollutant removal capacity and reduces the long-term performance of the material; channeling, where preferential flow paths reduce process efficiency; high costs associated with equipment, maintenance, and biosorbent replacement; and challenges in scaling up. Fluidized-bed systems address these drawbacks by maintaining the biosorbent in suspension by means of a controlled liquid or gas flow, thereby promoting efficient particle mixing and enhancing mass-transfer processes. This configuration reduces energy requirements (compared to mechanically stirred batch systems), increases resistance to fouling, and minimizes the occurrence of channeling within the reactor. The fluidization of the biosorbent increases the specific surface-to-volume ratio and improves mass transfer, resulting in more efficient pollutant removal. Moreover, fluidized-bed systems can be more readily scaled up, which makes them appropriate for diverse wastewater treatment applications [14,24].

Synthetic dyes are a group of chemical compounds extensively used as colorants across various industries due to the presence of chromophore groups, which impart vibrant and stable colors to materials [25]. Even at concentrations below 1 mg/L, these dyes act as micropollutants in aquatic environments. They include a wide type range, such as basic, reactive, anthraquinone, acidic, azo, diazo, and metal-complex dyes [26]. The persistence of these compounds in water can have long-term detrimental effects on aquatic ecosystems, including increased toxicity, reduced dissolved oxygen levels, heightened turbidity, and bioaccumulation in the food chain. The widespread use of synthetic dyes is driven by their low cost, ease of application, broad color variety, and stability, making them a significant source of environmental pollution compared to natural dyes. Exposure to textile dyes can also pose health risks to humans, causing skin and eye irritation, and even low concentrations in water may have carcinogenic effects [27]. Consequently, effective treatment of dye-containing wastewater is essential to protect both environmental and public health.

Among these, biosorption has emerged as an attractive approach, particularly through the valorization of residual microbial biomass generated as a by-product of biotechnological processes. In contrast to many synthetic or chemically modified adsorbents, whose production often requires considerable energy and chemical inputs, resulting in higher costs and environmental burdens, the biosorbent investigated in this study is obtained from spent brewing yeast, an abundant industrial by-product. The utilization of this residual biomass not only promotes waste valorization within a circular bioeconomy framework but also provides a cost-effective, renewable, and readily available material for the efficient removal of dyes from aqueous effluents. As an abundant and inexpensive biosorbent, this biomass can be immobilized in a sodium alginate matrix to improve its mechanical stability, facilitate handling, and enhance its applicability in continuous-flow wastewater treatment systems. In this context, the present study first examines biosorption under static (batch) conditions to characterize the behavior of the biosorbent and evaluate the effects of key parameters on pollutant removal. The findings derived from these batch experiments serve as the basis for the subsequent dynamic studies, where biosorption is assessed under continuous-flow conditions in a fluidized-bed column. This two-step approach allows the optimization of operational parameters for dynamic systems based on the fundamental understanding obtained from static experiments, linking laboratory-scale findings with continuous wastewater treatment applications. This research specifically examines the dynamic biosorption of O16 dye using immobilized residual *Saccharomyces pastorianus* biomass, a by-product of the beer industry. Kinetic models, including Yoon–Nelson, Bohart–Adams, Yan, and Clark, were applied to describe the biosorption behavior under continuous-flow conditions. The originality of this study is reflected in the combined investigation of Reactive Orange 16 biosorption using immobilized *Saccharomyces pastorianus* residual biomass under both batch and fluidized-bed continuous-flow conditions. By integrating process optimization, dynamic column modeling, and FTIR characterization, the work provides a comprehensive evaluation of the developed biosorbent and its potential application in dye-contaminated wastewater treatment.

The results of this study are intended to provide a basis for identifying optimal operating parameters for the development of industrial-scale treatment systems. Extending these findings to industrial wastewater applications will further demonstrate whether the examined microbial biosorbent possesses sufficient capacity and reliability for treating real effluents containing persistent organic pollutants in both static and dynamic regimes.

## 2. Materials and Methods

### 2.1. Materials

Biosorbent—Residual biomass of *S. pastorianus*, a by-product of the brewing process supplied by Albrau (Onești, Romania), was transformed into a practical, easy-to-handle biosorbent by immobilization in sodium alginate, as previously described [24,28]. Briefly, a suspension containing 5% (d.w.) residual yeast biomass in 1.5% low-viscosity sodium alginate solution was extruded dropwise through a 750 μm nozzle into a 100 mM CaCl_2_ gelling bath, using a Büchi Encapsulator B-390 (Büchi Labortechnik AG, Flawil, Switzerland) under controlled conditions (air pressure 100 mbar, T = 45 °C, 500 V, 800 Hz). This process yielded uniform spherical beads with a diameter of approximately 1.5 mm and homogeneous internal biomass distribution. The preparation procedure and physicochemical characterization of the biosorbent granules (SEM, EDAX, pH_PZC_) have been described in detail in our previous works [24,28], revealing a porous morphology, elemental composition, and surface-charge properties consistent with the material’s suitability for adsorption applications.

**Figure 1:**
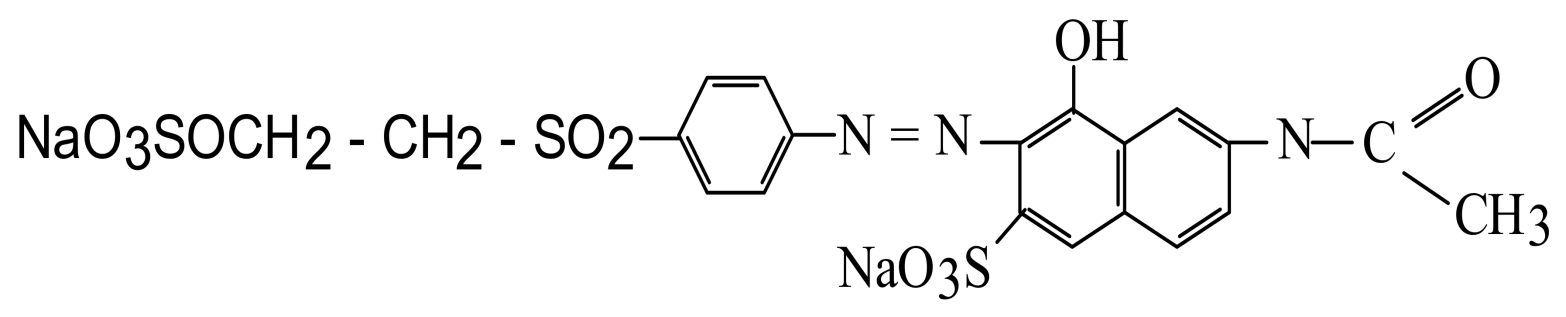
Chemical structure of reactive dye Orange 16.

Target dye—Orange 16 (O16—C.I. 18,097; and λ_max_ = 495 nm—Figure 1), an azo dye used in textile industries, was chosen as the synthetic model organic pollutant. This reactive dye is anionic and bifunctional and has a molecular weight of 617.54 g/mol.

A concentrated dye solution (725 mg/L) was initially prepared and subsequently diluted to produce synthetic aqueous working solutions with concentrations in the range of 21–232 mg/L.

### 2.2. Methods

#### 2.2.1. Quantitative Methods for Evaluating Dye Removal Efficiency

Solution samples collected at different time intervals were analyzed for residual dye concentration using a Shimadzu UV-1280 UV–VIS spectrophotometer (Shimadzu Corporation, Kyoto, Japan) at 495 nm. Dye concentrations were quantified from a calibration curve constructed within the linear range of the Lambert–Beer law. The retained dye content (q_e_) was used to assess this biosorbent’s sorption capabilities (Equation (1)):

(1) $$q_{e}=\frac{C_{0}-C_{e}}{G}\cdot V$$

where C_0_ and Ce indicate the initial and equilibrium dye concentrations (mg/L), G is the mass of biosorbent added (g), and V is the volume of solution processed (L).

The breakthrough curves for the selected O16 dye biosorption conditions, plotting effluent concentration (C_t_) or normalized concentration (C_t_/C_0_) against time (t), were applied to evaluate the performance of the prepared biosorbent.

#### 2.2.2. Batch-Mode Biosorption Studies

For the batch biosorption equilibrium experiments, 50 mL Erlenmeyer flasks were filled with 25 mL of dye solutions at varying initial concentrations (21–232 mg/L) and pH values (1–5), each containing 0.064–0.498 g of biosorbent (equivalent to 2.56–19.92 g/L). All assays were conducted at 25 °C. The pH of each solution was adjusted using 0.1 N NaOH or HCl and verified with a portable Hanna KL-009(I) pH meter (Hanna Instrument, Nusfalau, Romania). Additionally, based on the experimentally determined optimal pH and the biosorbent dosage required to achieve maximum dye retention, the maximum biosorption capacity (q_t_, mg/g) of the studied biosorbent was evaluated for the selected dye as the target organic pollutant at each time “t” considered in the experimental protocol. After equilibrium was attained, solution samples were collected and analyzed for residual O16 concentration.

#### 2.2.3. Dynamic Biosorption Procedure

**Figure 2:**
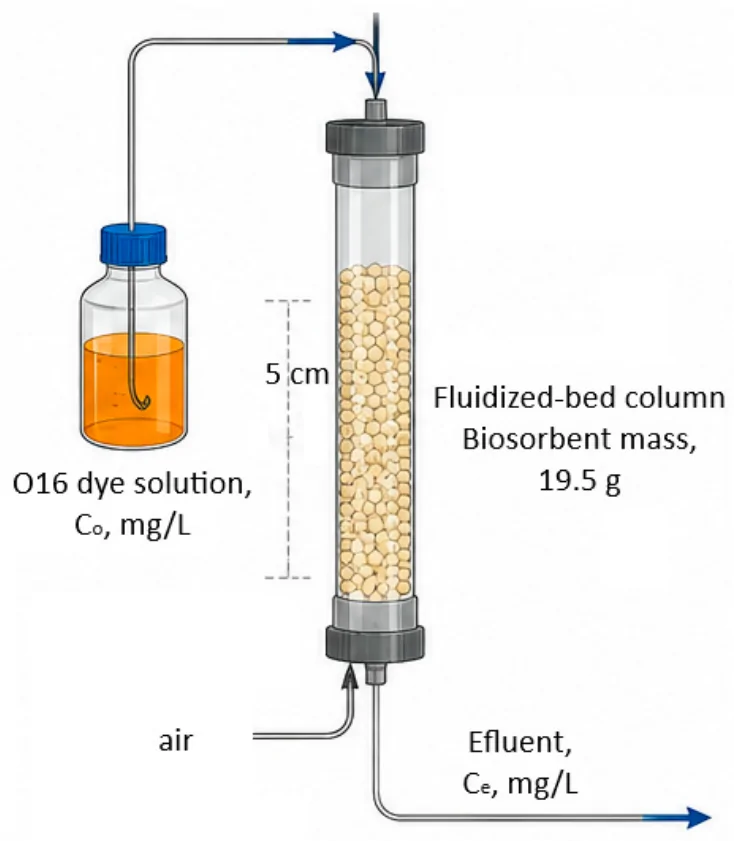
Diagram showing the O16 dye continuous biosorption procedure employing the prepared biosorbent.

For dynamic biosorption studies, a glass column of 15 cm length and 3 cm inner diameter was utilized. The column was filled with 19.5 g of biosorbent spheres, in the form of a layer 5 cm high (corresponding to a bed volume of approximately 35.3 cm^3^). The dye solution (with 87 mg/L concentration and pH = 2) was added to the top of the column using a feeding funnel in order to maintain a consistent, continuous-flow. The dye solution was transported through the column under gravitational conditions at flow rates of 3.8 and 8.5 mL/min. Periodic samples of the effluent were withdrawn every 10 min for subsequent spectrophotometric analysis (Figure 2). Air was introduced at the lower section of the column to induce biosorbent bead fluidization. The biosorption capacity in the continuous-flow experiments was calculated from the amount of dye retained by the biosorbent, determined from the concentration difference between the inlet and outlet streams over the treated solution volume and expressed per unit mass of biosorbent (mg/g).

All experiments were conducted under the following operational parameters:

(1) The feed temperature and pH were maintained at 25 °C and 2, respectively, in accordance with the optimal conditions identified in the batch biosorption investigation;
(2) Synthetic dye solutions prepared at the same initial concentration (87 mg/L) were introduced into the column containing a fluidized biosorbent bed (biosorbent mass, 19.5 g) at two distinct volumetric flow rates (Q_i_, i =1; 2). The applied flow rates were 3.8 mL/min and 8.5 mL/min, respectively.

Experiments were concluded once equilibrium was reached, which was defined either by stable dye concentrations over successive time periods or by the final dye concentration in the collected samples being equal to or higher than the initial concentration.

### 2.3. Dynamic Modeling of Biosorption Based on Experimental Data

The literature presents a variety of linear and non-linear numerical kinetic models to assess breakthrough behavior and the impact of independent variables on the dynamic adsorption system [14,15,16,19,21]. Several well-established models (Equations (2)–(5)) from the scientific literature were applied to the experimental data to determine the characteristic biosorption parameters of the dynamic biosorption system. Considering that for all applied models, C_0_ and C_t_ (mg/mL) define the dye concentrations at the column inlet and outlet, respectively, the parameters of significance in these equations are as follows:

- The Yoon–Nelson model [15,16,19] is founded on the premise that the rate at which adsorption probability decreases is proportional to both the probability of adsorption and the probability of breakthrough in the biosorbent bed:
  (2) $$\frac{C_{t}}{C_{0}}=\frac{1}{1+e^{\left(k_{YN}\cdot \tau -k_{YN}\cdot t\right)}}$$
  k_YN_ (min^−1^) is the model rate constant, τ (min) represents the time needed to reach 50% of the breakthrough concentration, and t (min) is the total biosorption operational time.
- In the Bohart–Adams model [14,15,19], the biosorption rate is assumed to be proportional to the amount or concentration of biosorbate in the bulk liquid phase and to the residual biosorption capacity of the biosorbent, disregarding the effects of axial dispersion.
  (3) $$\frac{C_{t}}{C_{0}}=\frac{e^{\left(k_{BA}\cdot C_{0}\cdot t\right)}}{e^{\left(k_{BA}\cdot C_{0}\cdot t\right)}+e^{k_{BA}\cdot N_{BA}\cdot Z/v}-1}$$
  where k_BA_ (mL/(mg·min)) denotes the model kinetic constant, N_BA_ (mg/mL) defines the maximum adsorption capacity per unit bed volume, Z (m) corresponds to the bed depth, v (m/min) refers to the superficial feed velocity, and t (min) is the biosorption operational time.
- The Clark model [15,19] is derived from the Freundlich isotherm and incorporates mass-transfer phenomena. The underlying assumptions are that external mass transfer controls the overall adsorption rate, that complete adsorbate removal occurs prior to breakthrough at the column outlet, and that the mass-transfer zone maintains a constant shape during column operation.
  (4) $$\frac{C_{t}}{C_{0}}={\left(\frac{1}{1+A_{ck}\cdot e^{\left(-r_{ck}\cdot t\right)}}\right)}^{\left(\frac{1}{n_{fr}-1}\right)}$$
  where A_ck_ (dimensionless) and r_ck_ (min^−1^) denote the model empirical constants. The parameter n_fr_ corresponds to the Freundlich exponent, and t (min) refers to the biosorption operational time.
- The Yan model [15,21] was introduced with the explicit goal of reducing the Thomas model’s adjustment error. The breakthrough behavior of the adsorption column was fitted using an empirical calibration approach:
  (5) $$\frac{C_{t}}{C_{0}}=1-\frac{1}{{\left(1+e^{(C_{0}\cdot Q\cdot t/q_{Y}\cdot m}\right)}^{k_{Y}}}$$
  where Q (mL/min) denotes the volumetric flow rate, and t (min) corresponds to the total biosorption operational time. The biosorbent mass is defined by m (g), while k_Y_ (dimensionless) and q_Y_ (mg/g) refer to the Yan rate constant and the maximum adsorption capacity of the biosorbent, respectively. The models and experimental data were compared using a nonlinear regression approach, in which the degree of agreement between model predictions and experimental values was assessed through regression coefficients (R^2^) approaching unity. In the present study, model performance was assessed based on the goodness of fit between experimental and predicted breakthrough curves. The objective was to identify the model that best describes the experimental behavior under the investigated operating conditions rather than to perform a comprehensive statistical validation of the applied models.

### 2.4. Physicochemical Characterization of Biosorbent

The biosorbent prepared by immobilizing residual *Saccharomyces pastorianus* biomass in a sodium alginate matrix was characterized before and after the biosorption process to evaluate the structural changes associated with dye uptake. Fourier Transform Infrared (FTIR) spectroscopy was employed to identify the functional groups present on the biosorbent surface that may participate in the biosorption of dye molecules. To elucidate the interaction mechanism between the biosorbent and the dye, FTIR spectra were recorded both before and after the biosorption process. The comparison of the spectra enabled the identification of functional groups involved in dye binding as well as the assessment of structural modifications induced by biosorption. Spectra were acquired using a Bruker Vertex 70 FTIR spectrometer (Bruker, Karlsruhe, Germany) equipped with an attenuated total reflectance (ATR) accessory, over the spectral range of 4000–400 cm^−1^, with a resolution of 2 cm^−1^ and 32 scans at room temperature.

## 3. Results and Discussion

The removal of persistent organic pollutants, specifically Orange 16, from aqueous solutions was investigated using biosorbents derived from residual microbial biomass immobilized in polymer matrices, under both static and dynamic conditions. In the static system, key operational parameters, including, pH, initial dye concentration, and biosorbent dosage, were evaluated.

### 3.1. Batch Regime Study Results on Orange 16 Dye Removal by the Prepared Biosorbent

**Figure 3:**
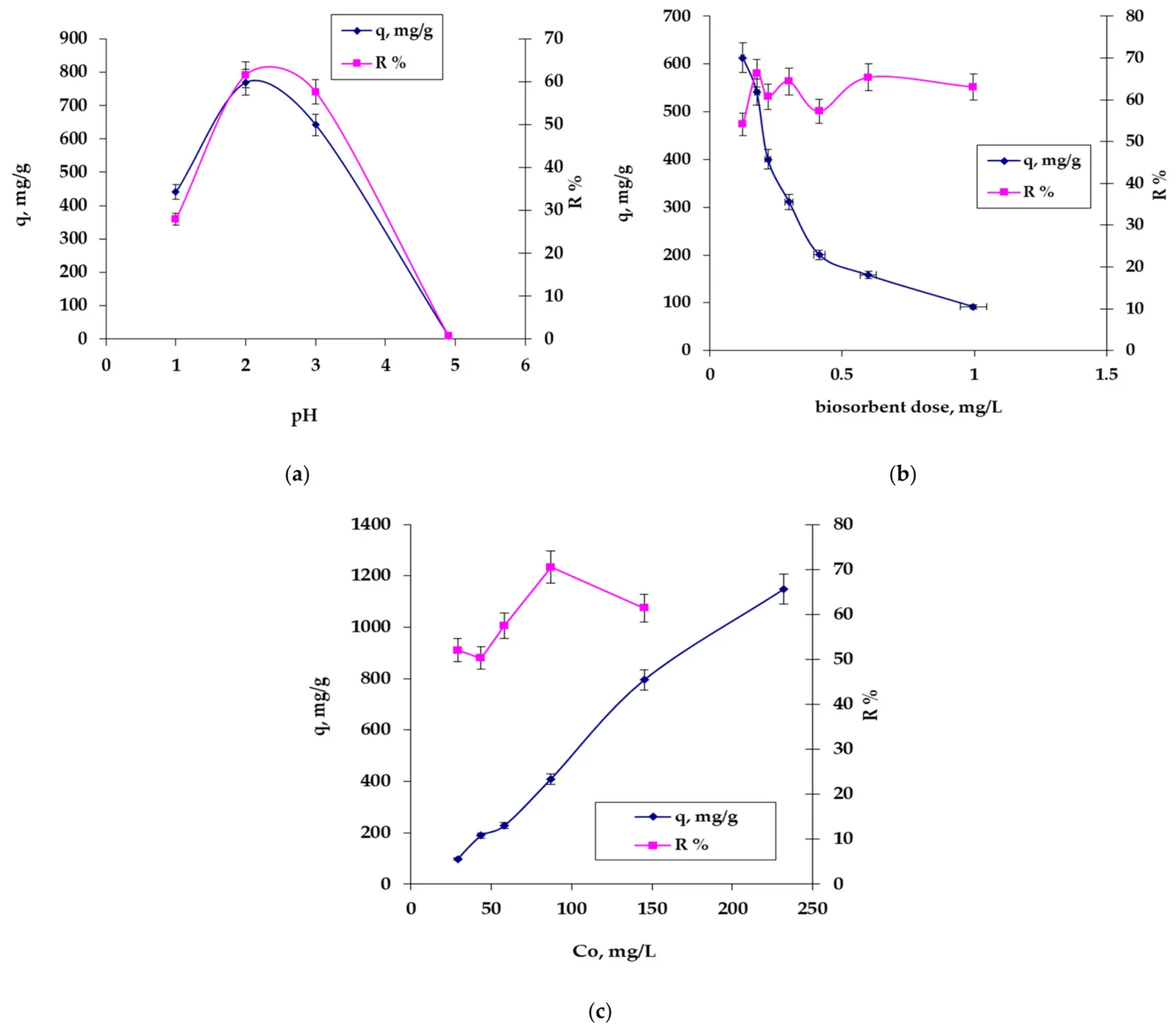
Operational parameters influencing the biosorption process of the Orange 16 dye onto the biosorbent: (**a**) the influence of pH (experimental conditions: C_0_ = 145 mg/L; biosorbent dose = 2.4 g/L with 5% d.w.); (**b**) the influence of biosorbent dose (experimental conditions: C_0_ = 145 mg/L; pH = 2); (**c**) the influence of initial dye concentration (experimental conditions: biosorbent dose = 2.4 g/L with 5% d.w. and pH = 2). Note: The biosorption capacity was calculated based on the biomass content of the granules.

The first part of the study was conducted to determine the operating conditions that maximize the biosorption capacity by examining the effects of selected operational parameters (pH, biosorbent dose, initial dye concentration) on the biosorption of Orange 16 dye on the studied biosorbent. The results are summarized in Figure 3.

Analysis of the data from Figure 3 leads to the following key conclusions:

- Biosorption depends on the pH of the aqueous solution (Figure 3a), as it affects both the surface charge of the biosorbent (via ionization of functional groups) and the ionic form of the dye in the solution (conversion of sulfonate groups to their sodium salt form). Considering the pH_PZC_ value of 5.4 for the studied biosorbent [29] and the molecular structure of the selected dye, the effect of pH on biosorption was investigated within the acidic range of 1–5. This pH-dependent behavior can be explained by the electrostatic interaction between the biosorbent surface and the dye molecule. Reactive Orange 16 is an anionic azo dye with two ionizable acidic groups (–SO_3_^−^ and –O–SO_3_^−^), which remain fully dissociated and negatively charged throughout the pH range investigated. Given the pH_PZC_ value of 5.4 for the biosorbent [29], the surface carries a net positive charge at pH values below this point, due to protonation of surface functional groups, whereas at pH values above 5.4 the surface becomes net negatively charged. At pH = 2, the pronounced protonation of the biosorbent surface generates a strong positive charge, which favors electrostatic attraction toward the anionic sulfonate groups of the dye and accounts for the maximum biosorption capacity observed at this pH. As the pH increases toward 5, the degree of surface protonation progressively decreases, weakening the electrostatic attraction between the biosorbent and the dye and thereby explaining the gradual decline in biosorption efficiency. Notably, the adsorption capacity (q) value at pH = 1 was lower than that at pH = 2, a decrease attributed to the markedly higher H^+^ concentration at pH = 1, which competes with the dye anions for the same binding sites on the biosorbent surface, thereby limiting their accessibility despite the more favorable surface charge.
- Figure 3b shows a decrease in biosorption capacity and an increase in R % values from 613.168 mg/g (54.13%) to 91.834 mg/g (63.08%) with increasing biosorbent dose from 0.126 g/L to 0.996 g d.w./L. This trend can be attributed to the aggregation of biosorbent particles at higher dosages, which reduces the effective surface area available for dye adsorption and may lead to partial overlapping of binding sites, thereby lowering the biosorption capacity per unit mass.
- From Figure 3c it is evident that the biosorption capacity increases with the initial dye concentration in the aqueous solution until the biosorbent reaches its saturation capacity. Such behavior is likely due to the greater driving force for mass transfer at higher dye concentrations, which enhances the interaction between dye molecules and the available binding sites on the biosorbent. Once the available sites are fully occupied, the biosorption capacity reaches a plateau, indicating that the biosorbent has attained its maximum uptake capacity.

To determine the maximum biosorption capacity under the established conditions, the experimental data were analyzed using the Langmuir isotherm model. The model is expressed both in its general form and in the linearized form (Equation (6a,b)):

(6a) $$q=\frac{K_{L}\cdot C\cdot q_{0}}{1+K_{L}\cdot C}$$

and the linearized form

(6b) $$\frac{1}{q}=\frac{1}{q_{0}}+\frac{1}{K_{L}\cdot q_{0}}\cdot \frac{1}{C}$$

where q_0_ (mg/g) represents the maximum biosorption capacity, q (mg/g) denotes the amount of solute biosorbed at equilibrium, and K_L_ (L/mg) corresponds to the Langmuir affinity constant, related to the binding energy of the solute onto the biosorbent surface.

The Langmuir isotherm parameters, determined from the slope and intercept of the linearized Langmuir plot (1/q = f s(1/C), are q_0_ = 1000 mg/g and K_L_ = 0.0119 L/g. The Langmuir constant value indicates a favorable interaction between Orange 16 molecules and the active sites of the biosorbent. Under the optimum acidic conditions (pH = 2), the biosorbent surface becomes positively charged due to protonation of amino and other functional groups, promoting strong electrostatic attraction toward the anionic sulfonate groups of the dye. This behavior is consistent with the high dye removal efficiencies observed experimentally. The maximum adsorption capacity predicted by the Langmuir model reflects the theoretical monolayer capacity of the biosorbent under equilibrium conditions and should therefore be interpreted with caution. Such a value does not necessarily correspond to the adsorption capacity attained under practical operating conditions but rather represents the capacity estimated from model extrapolation based on the experimental equilibrium data. The high q_max_ value may be attributed to the large number of available binding sites provided by the immobilized microbial biomass and to the favorable electrostatic interactions established between the dye molecules and the biosorbent surface under strongly acidic conditions.

The Dubinin–Radushkevich (D–R) model (Equations (7a,b)–(9)) was employed to assess the nature of the biosorption process, physical or chemical, based on the adsorption energy (E). Adsorption energies below 8 kJ/mol indicate a physical adsorption mechanism, while values between 8 and 16 kJ/mol indicate an ion-exchange mechanism [29,30,31]. This approach provides insight into the underlying interactions governing dye uptake by the biosorbent:

(7a) $$q=q_{0}\exp\left(-\beta \cdot \varepsilon^{2}\right)$$

and the linearized form

(7b) $$\ln q=\ln q_{0}-\beta \varepsilon^{2}$$

(8) $$\varepsilon =RTln\;\left(\frac{C+1}{C}\right)$$

(9) $$E=\frac{1}{\sqrt{2\beta }}$$

where q_0_ (mmol retained dye/g biosorbent) defines the maximum biosorption capacity, while C (mmol dye/L) represents the equilibrium dye concentration in solution; the Polanyi potential is denoted by ε, and β (mol^2^/kJ^2^) corresponds to the activity coefficient associated with the mean biosorption energy; E (kJ/mol) refers to the mean free energy of biosorption; R is the universal gas constant (J/(mol·K)); and T (°K) denotes the absolute temperature.

The biosorption energy (E) determined using Equations (7a,b)–(9) was 6.428 kJ/mol, a value that suggests that the biosorption process proceeds according to a physical mechanism.

### 3.2. Continuous-Flow Biosorption Study

The efficiency of biosorption in a fluidized bed for removing Orange 16 dye from aqueous solution using biosorbent beads (*S. pastorianus*/alginate matrix) is strongly dependent on the feed flow rate. Effective adsorption can only occur when the flow rate ensures sufficient contact time between the dye solution and the biosorbent surface, thereby enabling optimal mass transfer and achieving maximum biosorption capacity prior to bed saturation. The biosorption capacity of Orange 16 in the fluidized-bed column at time *t* (*q_t_*, mg/g) was calculated according to Equation (10):

(10) $$q_{t}=\frac{Q\cdot C_{0}}{m\cdot 1000}\cdot \int_{t=0}^{t=t_{s}}\left(1-\frac{C_{t}}{C_{0}}\right)dt$$

where *Q* is the flow rate (mL/min), *C*_0_ and *C_t_* are the influent and effluent concentrations (mg/L) of Orange 16 at time *t*, *t_s_* is the saturation time (min) and *m* (g) is the mass of the biosorbent packed in the column.

**Figure 4:**
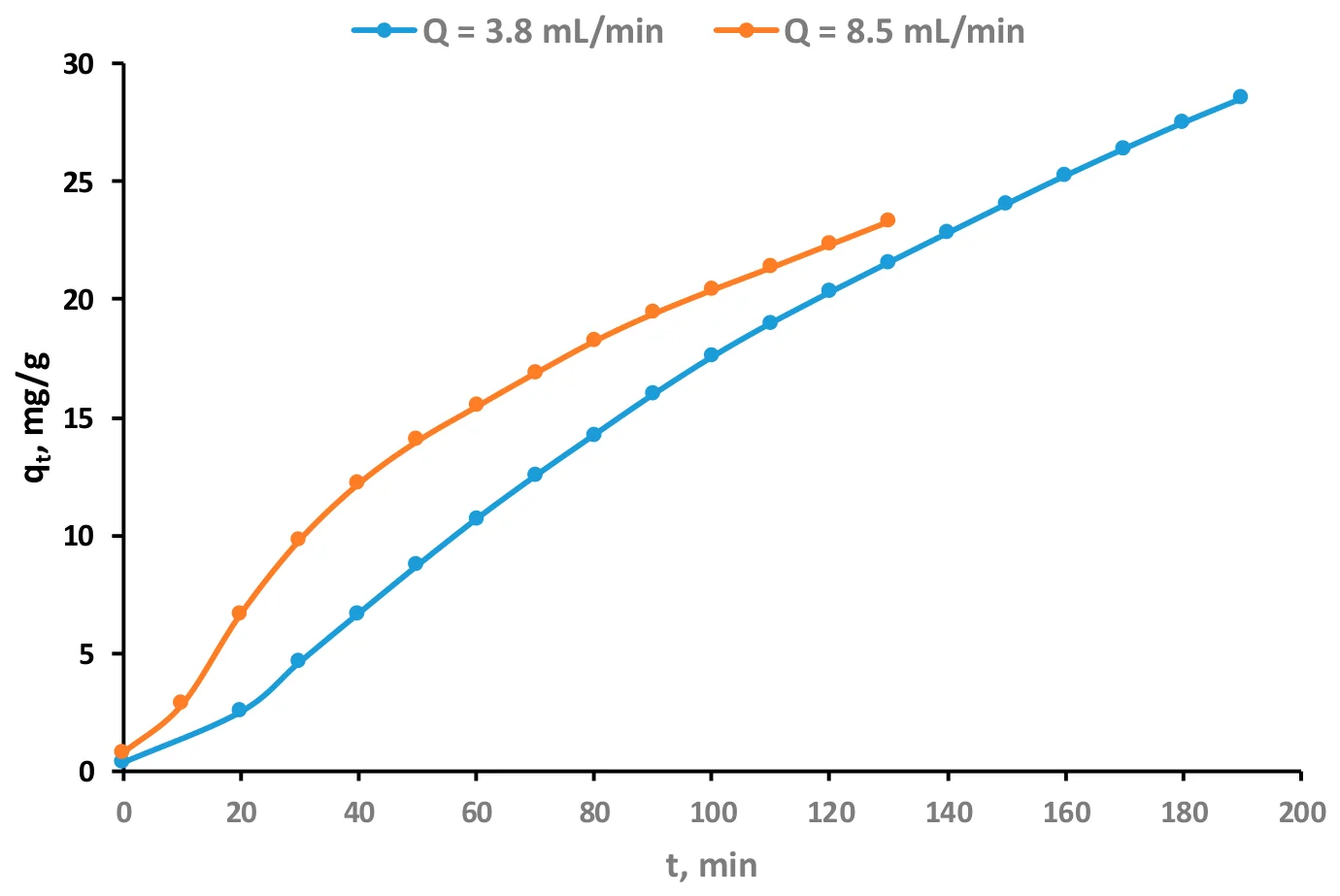
Effect of feed flow rate on the biosorption of O16 dye in a fluidized-bed system using the prepared biosorbent (q_t_—biosorption capacity at time t, mg/g; Q—flow rate, mL/min) Note: The biosorption capacity was calculated based on the biomass content of the granules.

The influence of the initial feed flow rate on adsorption performance was investigated for two inlet flow rates, 3.8 and 8.5 mL/min, and a fixed-bed biosorbent mass of 19.5 g, with bead diameters of 1.5 mm and an initial dye concentration of 87 mg/L. The resulting sorption behaviors at the two flow rates are presented in Figure 4, and clearly demonstrate the significant effect of feed flow rate on the biosorption of Orange 16 dye in a fluidized-bed system.

At the higher flow rate (8.5 mL/min), the biosorption capacity (qt) increased more rapidly during the initial stage of the experiment because a larger amount of dye was supplied to the column per unit time (Figure 4). However, the shorter residence time of the solution within the biosorbent bed reduced the contact time between the dye molecules and the available adsorption sites. As a result, although dye uptake was initially faster, the maximum biosorption capacity achieved at 8.5 mL/min was lower (approximately 23–24 mg/g) than that obtained at the lower flow rate of 3.8 mL/min, where the final capacity exceeded 28 mg/g. This behavior indicates that longer residence times favor more effective utilization of the biosorbent bed and consequently lead to higher overall biosorption efficiency.

The results showed that the maximum biosorption capacity of immobilized residual *S. pastorianus* biomass for the O16 anionic dye ranged from 23 to 28 mg/g in the continuous-flow experiments, compared to approximately 1000 mg/g in the batch regime. These results indicate that the batch system clearly outperformed the fixed-bed column system in terms of biosorption capacity. The extended and continuous contact between the biosorbent and the dye solution, along with the high specific surface area of the biosorbent, likely contributed to the superior performance of the batch biosorption system, even though the concentration gradient gradually decreased over time.

The marked difference between the adsorption capacity obtained in batch equilibrium experiments and that observed under continuous-flow conditions can be further explained by the fundamental differences between the two operating modes. In batch systems, the biosorbent is allowed to remain in contact with the dye solution until adsorption equilibrium is approached, providing sufficient time for dye molecules to diffuse from the bulk solution to the external surface and subsequently into the internal structure of the biosorbent particles. Under these conditions, a large fraction of the available binding sites can be occupied, resulting in high equilibrium adsorption capacities. In contrast, adsorption in the fluidized-bed column is inherently a non-equilibrium process. The residence time of the dye solution inside the column is limited by the imposed flow rate, and part of the adsorbate leaves the system before complete equilibration with the biosorbent is achieved. In addition, mass-transfer limitations may occur at several stages, including transport through the external liquid film surrounding the particles and intraparticle diffusion within the alginate-immobilized biomass beads. These resistances slow the migration of dye molecules toward internal active sites and reduce the overall extent of adsorbent utilization during the dynamic operation. Furthermore, adsorption in the column takes place through the progressive formation and movement of a mass-transfer zone rather than through uniform saturation of the entire biosorbent bed. Consequently, parts of the adsorption sites remain only partially utilized when breakthrough occurs, leading to lower apparent adsorption capacities than those measured under equilibrium batch conditions. The observed capacity of 23–28 mg/g should therefore be regarded as a dynamic operating capacity reflecting hydrodynamic constraints, residence time limitations, and mass-transfer effects, whereas the Langmuir capacity obtained in batch experiments represents the maximum equilibrium uptake achievable under idealized static conditions.

**Figure 5:**
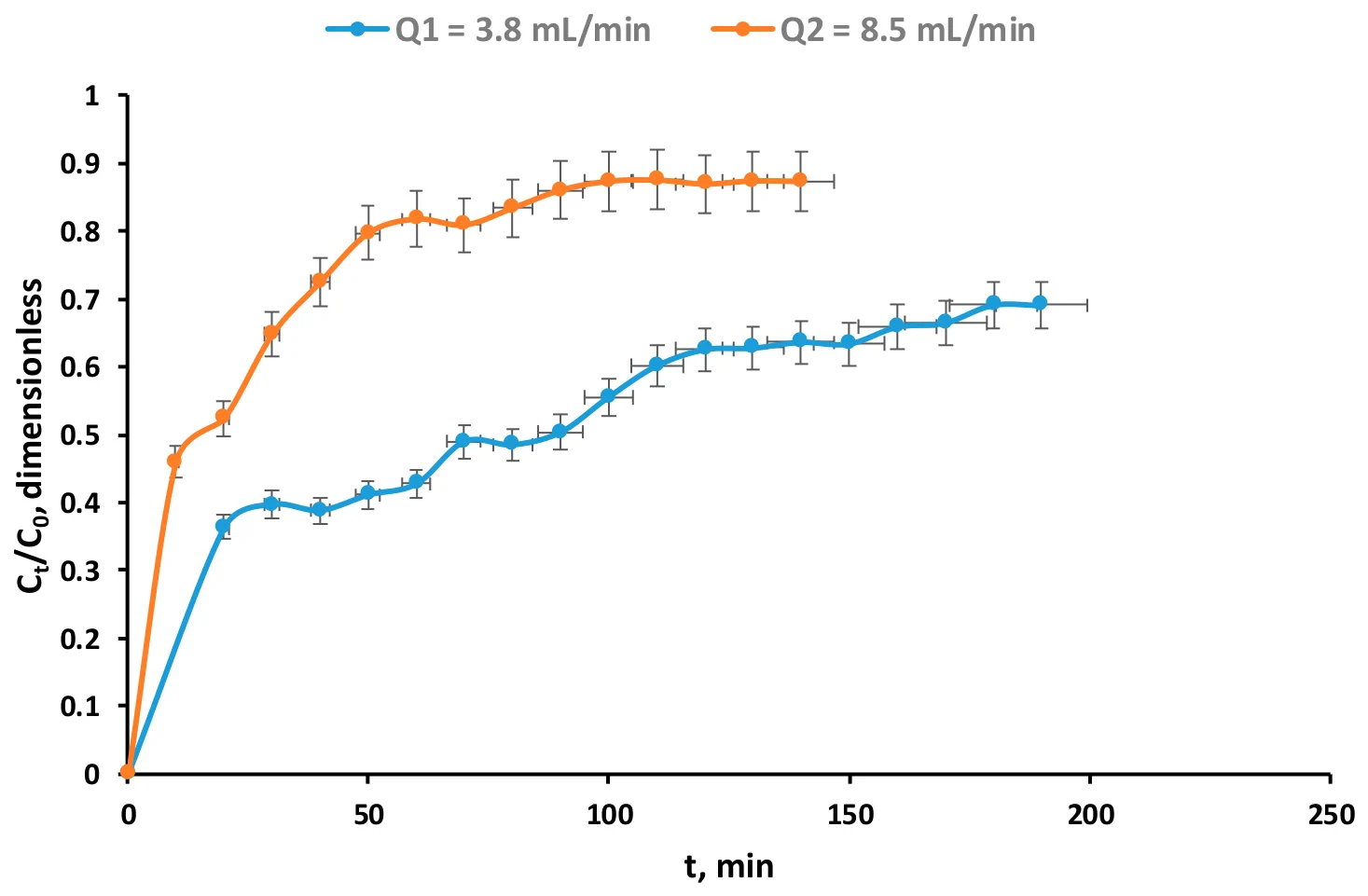
Breakthrough curves for the biosorption of O16 dye in a continuous fluidized-bed column packed with prepared biosorbent (experimental conditions: biosorbent-bed mass = 19.5 g; beads size = 1.25 mm; C_0_ = 87 mg O16/L; flow rates: Q_1_ = 3.8 mL/min and Q_2_ = 8.5 mL/min).

The breakthrough curves for O16 dye retention under continuous fluidized-bed operation conditions on a column packed with biosorbent are displayed in Figure 5. These curves are represented as the ratio of dye concentration at time t to the initial concentration (C_t_/C_0_—dimensionless; C_0_—mg/L, C_t_—mg/L) plotted against adsorption time (min), which is the standard approach for evaluating column performance in dynamic biosorption systems [32,33]. The shape of the breakthrough curve provides essential information about the loading behavior of the dye within the column and the progression of the biosorption process, including mass transfer and saturation phenomena [34].

At the higher flow rate (8.5 mL/min), breakthrough occurs rapidly, with C_t_/C_0_ reaching approximately 0.8 within the first 60 min, indicating faster saturation of the biosorbent due to the shorter contact time between the dye and the biosorbent surface. Conversely, at the lower flow rate (3.8 mL/min), breakthrough is delayed, and the column maintains a lower effluent concentration for a longer period, achieving C_t_/C_0_ ≈ 0.7 after 180 min. This behavior confirms that lower flow rates favor extended contact time and better utilization of the available binding sites, while higher flow rates limit overall retention despite delivering a higher initial mass flux of dye.

Similar breakthrough curve behaviors have been reported for organic dyes in continuous systems. Muralikrishnan and Jodhi (2021) observed that for Reactive Orange 16 in packed-bed columns, breakthrough time decreased significantly as flow rate increased from 0.3 L/h to 0.6 L/h, confirming the critical role of flow rate in column performance [32]. Obaid et al. (2017) also reported that higher flow rates shorten breakthrough time and reduce column efficiency for RO16 removal using kenaf-based adsorbents [35]. Jagaba et al. (2025) demonstrated that engineered biocomposites for dye removal exhibit steep breakthrough curves at high flow rates, confirming that external mass transfer limitations dominate under such conditions [36]. Cundari et al. (2022) showed that for dye removal of Jumputan wastewater, breakthrough time decreased significantly as flow rate increased from 10 to 30 mL/min, confirming the negative effect of high flow on column saturation [37]. Dastgerdi et al. (2023) observed that for Indigo Carmine, breakthrough curves became steeper at higher flow rates, reducing adsorption efficiency [38]. Hariharan et al. (2023) reported earlier breakthrough for azo dyes (RO16, RB-120) at increased flow rates, consistent with external mass transfer limitations [34]. Nabila et al. (2024) demonstrated that for Methylene Blue and Crystal Violet, lower flow rates extended breakthrough time and improved column utilization [39]. Finally, Tran et al. (2024) proposed a logistic-exponential model for Methylene Blue adsorption on biochar, highlighting that flow rate and bed height strongly influence breakthrough curve shape [40].

Furthermore, studies on biosorption systems using immobilized biomass in alginate [24,41,42] emphasize that contact time and hydrodynamic conditions strongly influence breakthrough behavior, a pattern consistent with the present results, which show that flow rate is essential to balance rapid dye removal with maximum biosorbent utilization in fluidized-bed systems.

### 3.3. Kinetic Modeling of the Biosorption Process in a Fluidized-Bed System

To determine the characteristic behavior of the dynamic biosorption system, the experimental data were analyzed using well-established kinetic models from the literature: Yoon–Nelson, Bohart–Adams, Clark, and Yan: Equations (2)–(5). These models were applied to describe the adsorption kinetics of Orange 16 dye onto the biosorbent under continuous fluidized-bed conditions. For each model, the experimental data were fitted using their nonlinear forms to ensure accurate parameter estimation and interpretation of adsorption dynamics. The evaluation focused on breakthrough characteristics such as breakthrough time, saturation time, and column efficiency, which are critical indicators of system performance. By analyzing these parameters, the effectiveness of the fluidized-bed system was assessed under the studied operational conditions, providing insights into mass transfer limitations and adsorbent utilization.

**Figure 6:**
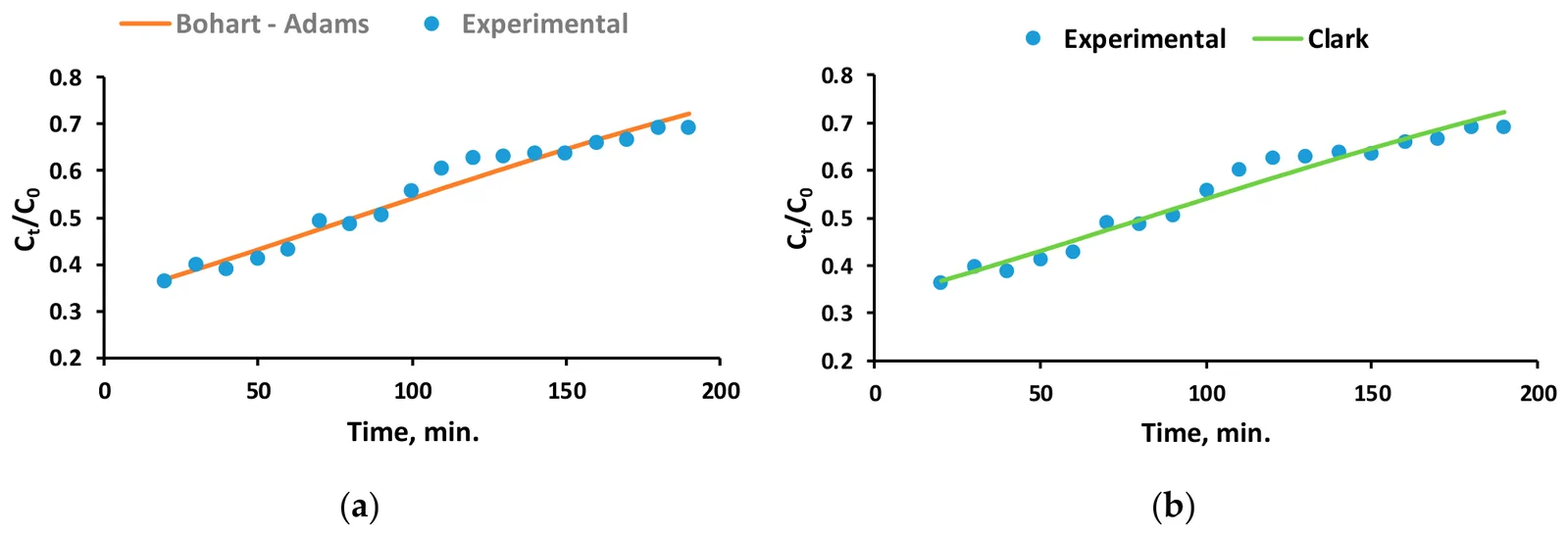

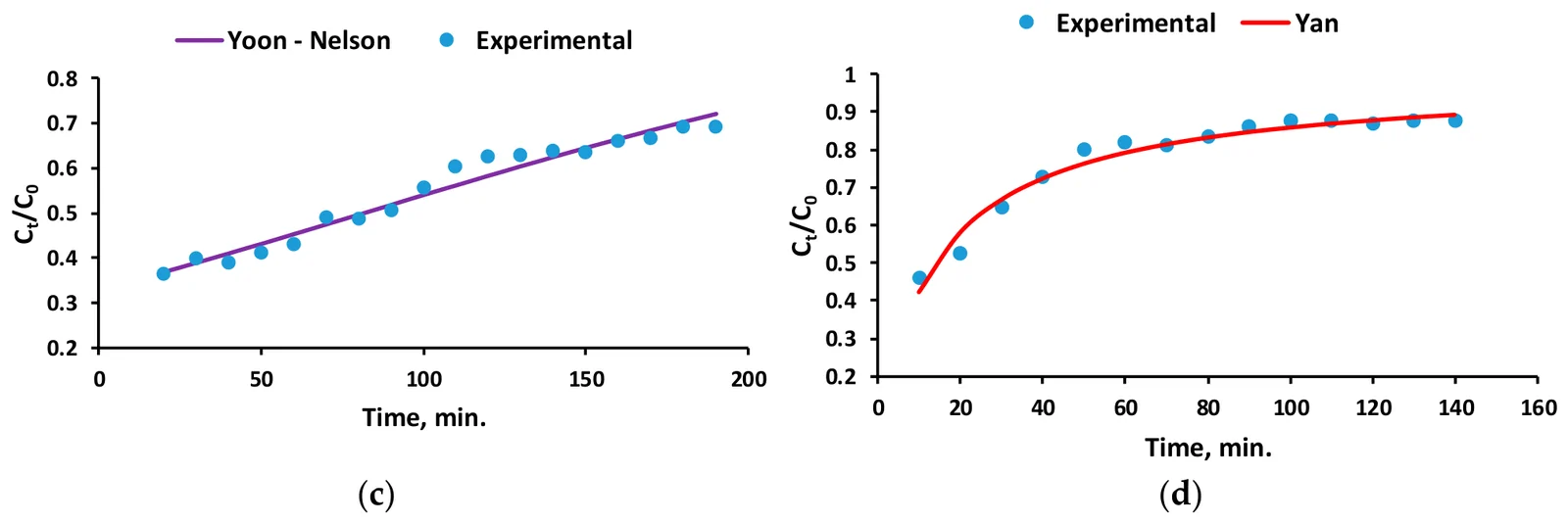
Breakthrough curves for O16 dye biosorption onto prepared biosorbent in the dynamic system, comparing experimental data with predictions from the Yoon–Nelson, Bohart–Adams, Clark, and Yan models. (**a**–**c**) correspond to a flow rate of 3.8 mL/min (inlet concentration: 87 mg/L; biosorbent amount: 19.5 g); (**d**) represents a flow rate of 8.5 mL/min under identical working conditions. Note: The figure includes only the models fitted for each specific flow rate.

The analysis of the nonlinear model equations revealed distinct behaviors at different flow rates. At a lower flow rate (3.8 mL/min—Table 1, Figure 6a–c), the Yoon–Nelson, Bohart–Adams, and Clark models provided excellent fits (R^2^ = 0.9638), indicating that these models effectively describe adsorption dynamics under conditions of extended contact time. A comparable behavior was reported by Hariharan et al. (2023) and Cundari et al. (2022), where packed-bed adsorption of azo dyes exhibited strong agreement with the Yoon–Nelson and Bohart–Adams models at low flow rates [34,37]. This similarity is likely due to the longer residence time, which enhances dye diffusion and utilization of adsorption sites. The good model performance observed in the present study therefore supports the interpretation that adsorption kinetics at 3.8 mL/min are governed by a more effective interaction between external mass transfer and intraparticle diffusion processes.

In contrast, at the higher flow rate (8.5 mL/min—Table 2 and Figure 6d), the Yan model achieved the best fit (R^2^ = 0.9676), while other models showed reduced accuracy (R^2^ < 0.88). The superior performance of the Yan model at the higher flow rate suggests that the adsorption process was influenced by non-equilibrium conditions associated with rapid transport of the dye solution through the biosorbent bed. Unlike the Bohart–Adams and Yoon–Nelson models, which are based on more simplified assumptions regarding adsorption kinetics and breakthrough behavior, the Yan model is better able to describe steep breakthrough curves and rapid bed saturation. The excellent fit obtained at 8.5 mL/min therefore indicates that external mass-transfer resistance and limited residence time played an important role in controlling dye uptake. Under these conditions, adsorption sites are not fully utilized before breakthrough occurs, and the adsorption front moves rapidly through the column. Consequently, thees results suggest that the dynamic biosorption process becomes increasingly governed by hydrodynamic and mass-transfer effects as the flow rate increases.

Similar observations were made by Dastgerdi et al. (2023) for Indigo Carmine and by Tran et al. (2024) for Methylene Blue, where Yan’s model was more suitable for predicting rapid breakthrough and saturation under high flow conditions, reflecting the dominance of external mass transfer constraints [38,39,40]. In these studies, the superior performance of the Yan model was attributed to its ability to account more effectively for steep breakthrough curves associated with rapid bed saturation. The agreement with our results suggests that external mass-transfer limitations become important as flow rate increases. Therefore, the Yan model appears particularly suitable for describing the breakthrough curves obtained in the present fluidized-bed system at 8.5 mL/min.

These comparisons confirm that model applicability is strongly influenced by hydrodynamic conditions: multi-parameter models like Yan perform better when adsorption is controlled by external diffusion, whereas simpler models (Yoon–Nelson, Bohart–Adams) are reliable under conditions favoring internal diffusion and longer contact times.

The comparative analysis of the kinetic parameters for applied kinetic models also reveals distinct behaviors as a function of feed flow rate.

The Bohart–Adams model, commonly employed to describe the initial phase of breakthrough curves, exhibited k_BA_ values of 0.10895 at 3.8 mL/min and 0.240277 at 8.5 mL/min. Lai et al. (2024) reported that the k_BA_ values increased from 0.444 to 1.393 mL/mg·min when flow rate increased during the adsorption of Metanil Yellow (MY) dye from contaminated water using a three-dimensional graphene oxide–chitosan biopolymer composite (3D-GCB) in a continuous fixed-bed system [42]. The appropriate fitting of the fluidized-bed column data with the Bohart–Adams model suggested that the earlier portion of the column was constrained by the transfer of mass in the external film surrounding the adsorbent. However, the decrease in R^2^ from 0.9638 to 0.8720 confirms literature observations regarding the sensitivity of this model to increasing flow rate [41,42,43].

The Clark model, which integrates the Freundlich isotherm into breakthrough curve prediction, exhibited a strong correlation at a low flow rate (R^2^ = 0.9638) but decreased accuracy at a higher flow rate (R^2^ = 0.8721). Hu et al. (2020) similarly noted that the Clark approach performs well under moderate conditions but loses predictive stability when hydrodynamic parameters vary in fixed-bed adsorption systems [44]. The decline in Clark model accuracy at a higher flow rate may be attributed to deviations from the model assumptions regarding the stability of the mass-transfer zone and adsorption equilibrium. Similar behavior reported in the literature indicates that hydrodynamic effects become important under rapid-flow conditions, reducing the applicability of models originally developed for more uniform adsorption regimes.

Conversely, the Yan model exhibited an excellent fit at higher flow rates, achieving R^2^ = 0.9676. This finding aligns with recent studies emphasizing the good fit of the Yan model for predicting breakthrough curves in dye adsorption under continuous operation and different hydrodynamic conditions. For instance, Hazarika et al. (2025) reported a maximum adsorption capacity of 70.38 mg/g for removing Acid Yellow 25 dye using a novel adsorbent from industrial tea waste [45]. Although the adsorption capacities reported by Hazarika et al. were considerably higher than those obtained in the present work, this difference can be attributed to the distinct nature of the adsorbent material, dye characteristics, and operating conditions. Nevertheless, both studies demonstrate that the Yan model remains robust across different adsorption systems, supporting its suitability for predicting breakthrough behavior under dynamic operating conditions.

For the Yoon–Nelson model, parameter evolution followed the expected pattern: k_YN_ increased from 0.008778 to 0.029094, while the breakthrough time τ decreased from 81.9662 min to 4.8446 min. Similar results were reported by Rivera-Arenas et al. (2023), who investigated the use of organoclay/alginate hydrogels for removing Acid Yellow 23 in a fixed-bed column [46]. When the flow rate increased from 1 mL/min to 5 mL/min, the obtained values for k_YN_ were 0.0263 and 0.0568, respectively, while the corresponding values for τ were 140.2932 min and 21.3060 min.

Overall, these findings confirm that the Yan model provides more stable predictions under a higher flow rate, whereas Bohart–Adams, Clark and Yoon–Nelson exhibit greater sensitivity to hydrodynamic changes.

Considering the aspects discussed above, it can be concluded that the evaluated models are suitable for predicting the biosorption of Orange 16 dye onto the prepared biosorbent.

### 3.4. Evaluation of Functional Groups Involved in Orange 16 Biosorption by FT-IR Spectroscopy

**Figure 7:**
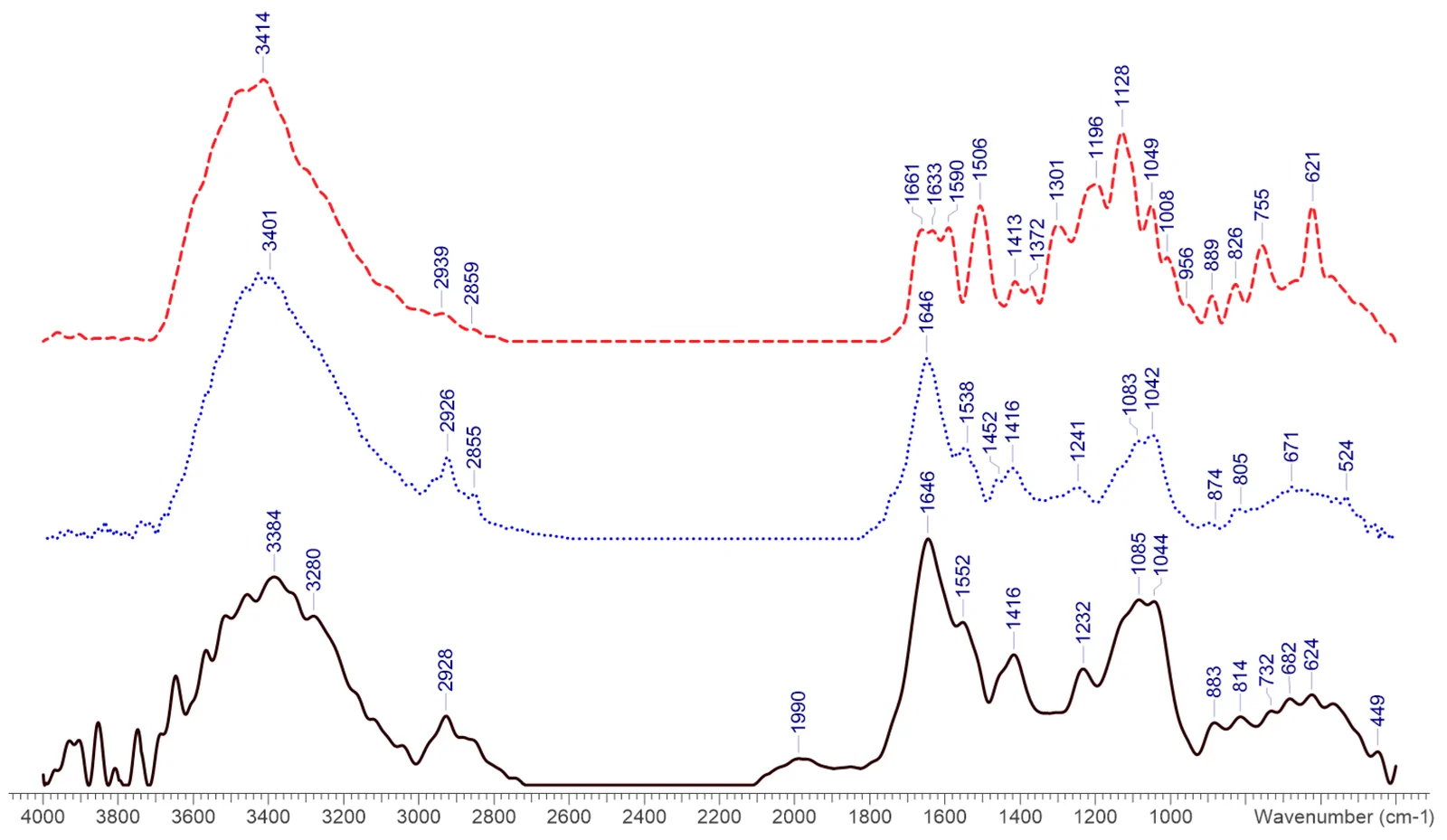
FT-IR spectra of the biosorbent before (black line) and after (blue line) biosorption and of reactive dye Orange 16 (red line).

The FTIR spectra obtained for the biosorbent before and after the reactive-dye biosorption process as well as the spectrum of the studied dye Orange 16 are presented in Figure 7.

The spectrum of the organic dye (red line in Figure 7) includes several peaks, and assignments to functional groups were made: (1) ~3400 cm^−1^ in the hydroxyl and amine region, and stretching (O-H hydroxyl groups) and/or N-H in amine/amide groups; (2) 2939 and 2859 cm^−1^ aliphatic hydrocarbons C-H in -CH_2_- and -CH_3_; (3) carbonyl and conjugated double bonds (C=O (1661 cm^−1^) and C=C (1633, 1590, 1506 cm^−1^)) and possible N=N stretching; (4) Fingerprint Region (1400–600 cm^−1^) that includes [47] 1301 cm^−1^ corresponding to aromatic C–N stretching vibrations; 1196, 1128, 1049, and 1008 cm^−1^ possibly attributed to sulfonate groups (-SO_3^−^_) or sulfonic acid sodium salts and symmetric S=O stretching [48]; 755 and 621 cm^−1^ attributed to out-of-plane aromatic C–H bending, indicating the substitution pattern on the benzene rings.

In the spectra of biomass (line black in Figure 7) and loaded (after biosorption) biomass (blue line in Figure 7): (1) the appearance of a sharper 2855 cm^−1^ peak potentially confirms the presence of the dye’s hydrocarbon backbone; (2) at 1646 cm^−1^, the backbone carbonyls of the biomass proteins maintain their frequency but overlap with the dye’s conjugated system; (3) the shift from 1552 to 1538 cm^−1^ of N-H and C-N bonding represents potential evidence that biomass cell-wall proteins are actively binding the dye [49]; (4) and the new peak at 1452 cm^−1^ receives a direct contribution from the aromatic skeleton of the adsorbed dye [50]. The broadened sulphonate profile (1196, 1128, 1049, and 1008 cm^−1^) suggests that the polysaccharide matrix of microbial biomass absorb the dye’s bands [51].

The FTIR results indicate that Orange 16 uptake is governed mainly by electrostatic interactions between the negatively charged sulfonate groups of the dye and positively charged amino groups of the biomass surface under acidic conditions. The shift in the amide band (1552→1538 cm^−1^) demonstrates the participation of cell-wall proteins in dye binding, while changes in the sulfonate and aromatic vibration regions confirm the adsorption of Orange 16 onto the biosorbent surface. Hydrogen bonding involving hydroxyl, amide, and polysaccharide groups may further contribute to dye retention. The low adsorption energy obtained from the Dubinin–Radushkevich model (6.43 kJ/mol) suggests that the process is predominantly physical biosorption. Overall, the biosorption mechanism can be described as predominantly electrostatic binding of Orange 16 sulfonate groups onto protonated amino sites of the microbial biomass, assisted by hydrogen bonding and weak van der Waals interactions involving hydroxyl, amide, and polysaccharide functionalities from the alginate–biomass matrix.

### 3.5. Performance of the Studied Biosorbent Compared to Other Similar Materials

Although limited studies have reported dye adsorption in fixed-bed columns using residual microbial biomass as a biosorbent, a comparative overview of the available literature data is presented in Table 3.

The comparison presented in Table 3 indicates that the adsorption performance reported in the literature varies considerably depending on the type of biomass, immobilization strategy, target dye, and operating regime. Several biosorbents exhibited higher adsorption capacities than those obtained in the present continuous-flow study. However, direct comparison among studies is difficult because adsorption performance depends not only on the physicochemical characteristics of the biosorbent but also on key operational parameters, including influent dye concentration, solution pH, column dimensions, flow rate and bed height. The obtained results demonstrate the promising performance of the developed biosorbent in both batch and fixed-bed column biosorption systems. The main advantage of the proposed biosorbent is not solely its adsorption capacity but also its sustainable origin, as it is produced from residual brewing biomass that would otherwise require disposal. Furthermore, unlike many adsorbents derived from chemically modified materials, the developed biosorbent can be prepared through a relatively simple immobilization process. Overall, these findings confirm the potential of the proposed biosorbent as an efficient and sustainable material for dye removal and provide a solid basis for future investigations using real textile wastewater, where the effects of complex wastewater matrices and competing contaminants should be systematically evaluated.

## 4. Conclusions

The removal of persistent organic pollutants, specifically Orange 16, from aqueous solutions was investigated using biosorbents derived from residual microbial biomass immobilized in polymer matrices, under both static and dynamic conditions.

In batch system the results revealed that process efficiency is strongly dependent on operational parameters such as pH, biosorbent dosage, and initial dye concentration. Maximum uptake was achieved under acidic conditions (pH = 2), while higher dye concentrations enhanced biosorption until saturation was reached.

FT-IR characterization demonstrated that amino, amide, hydroxyl, and polysaccharide groups of the S. pastorianus/alginate biocomposite participate in Orange 16 biosorption. The adsorption mechanism is mainly governed by electrostatic attraction between protonated biosorbent sites and the dye sulfonate groups, while hydrogen bonding contributes to the stabilization of the biosorbent–dye complex. The FT-IR results, together with the low D–R adsorption energy, indicate that Orange 16 removal occurs predominantly through a physical biosorption mechanism.

In a dynamic regime the study demonstrated that feed flow rate significantly influences the biosorption performance of Orange 16 dye in a fluidized-bed system using immobilized *S. pastorianus* biomass in sodium alginate.

A higher flow rate (8.5 mL/min) accelerates initial biosorption of Orange 16 due to improved mass transfer but results in lower overall capacity (23–24 mg/g). A lower flow rate (3.8 mL/min) ensures a longer contact time, achieving a higher capacity (>28 mg/g).

Therefore, optimizing flow rate is critical to balance rapid uptake with maximum adsorption capacity in fluidized-bed systems for Orange 16 dye removal.

Flow rate was found to significantly influence biosorption performance, with lower flow rates favoring higher overall efficiency due to longer contact time between the dye and the biosorbent.

The kinetic modeling analysis highlights that the predictive accuracy of breakthrough models in fluidized-bed biosorption systems is highly dependent on hydrodynamic conditions. At the lower flow rate of 3.8 mL/min, the Yoon–Nelson, Bohart–Adams, and Clark models provided reliable fits (R^2^ = 0.9638), indicating their suitability for conditions with extended contact time. Conversely, at the higher flow rate of 8.5 mL/min, the Yan model achieved the best performance (R^2^ = 0.9676), demonstrating its robustness under high-flow conditions where rapid mass transfer dominates.

These results underscore the importance of selecting appropriate models based on hydrodynamic regime to ensure accurate performance evaluation and process optimization.

Beyond the specific results obtained for Orange 16 removal, this study demonstrates the potential of integrating residual microbial biomass valorization with continuous biosorption technologies for sustainable wastewater treatment. The combined evaluation of batch and fluidized-bed systems provides valuable insight into the transition from equilibrium laboratory studies to dynamic operating conditions that more closely resemble industrial practice. The findings also highlight the importance of hydrodynamic effects and mass-transfer limitations in determining the effective utilization of biosorbents under continuous-flow conditions.

From a practical perspective, the developed biosorbent offers a low-cost and environmentally friendly solution for dye-contaminated wastewater treatment while promoting the circular valorization of industrial biowaste. Its favorable performance in both batch and fixed-bed column systems demonstrates its potential for continuous wastewater treatment applications.

Future research should focus on evaluating the performance of the biosorbent in real textile effluents, investigating its regeneration and reuse potential, assessing long-term operational stability, and examining the scalability and techno-economic feasibility of industrial-scale implementation.

## Figures and Tables

**Table 1:** Dynamic modeling of the biosorption process in the case of the O16 dye with Q_1_ = 3.8 mL/min (C_0_ = 87 mg/L; biosorbent mass = 19.5 g).

Model	*k_BA_*	*N_BA_*	*r_ck_*	*A_ck_*	*q_Y_*	*k_Y_*	*k_YN_*	*τ*	*R* ^2^
Bohart–Adams	0.1009	0.5549	—	—	—	—	—	—	0.9638
Clark	—	—	0.0088	2.0534	—	—	—	—	0.9638
Yan	—	—	—	—	1.1544	0.7053	—	—	0.9242
Yoon–Nelson	—	—	—	—	—	—	0.0088	81.9662	0.9638

**Table 2:** Dynamic modeling of the biosorption process in the case of the O16 dye with Q_2_ = 8.5 mL/min (C_0_ = 87 mg/L; biosorbent mass = 19.5 g).

Model	*k_BA_*	*N_BA_*	*r_ck_*	*A_ck_*	*q_Y_*	*k_Y_*	*k_YN_*	*τ*	*R* ^2^
Bohart–Adams	0.2403	−22.4214	—	—	—	—	—	—	0.8721
Clark	—	—	0.0209	1.1066	—	—	—	—	0.8721
Yan	—	—	—	—	0.5313	0.9189	—	—	0.9676
Yoon–Nelson	—	—	—	—	—	—	0.0209	4.8446	0.8721

**Table 3:** Comparative assessment of the adsorption performance of the studied biosorbent and related materials reported in the literature.

Biomass/Support for Immobilization	Target Dye and Biosorption Performance	Ref.
*A. campestris* (fungi)	Sirius Blue K-CFN (azo dye) biosorption, obtaining improved uptake of dye molecules with increasing packing heights with a maximum of 97.32% biosorption.	[17]
Brewery-spent grains (microbial fermentation residue)	Reactive Blue 5G dye in batch and continuous-flow systems, obtaining a dye removal of 94.5% and optimum results in continuous systems with 4 g biosorbent and a flow rate of 2 mL/min. Dynamic capacity reported by Thomas/Yoon–Nelson models with R% > 90. The maximum biosorption capacity achieved was 83.42 mg/g.	[16]
*Thamnidium elegans* (fungi) immoblized in *Phragmites australis*	Biosorption yield of 96.51% and saturation biosorption capacities in the column mode of 104.58 in a Reactive Blue 49 dye solution and 70.98 mg/g in real wastewater samples.	[52]
*Saccharomyces pastorianus* residual biomass immobilized in sodium alginate	Brilliant Red HE-3B reactive dye/efficiency was obtained for a flow rate of 4 mL/min, an initial pollutant concentration of 51.2 mg/L, and a biosorbent amount of 20 g.	[24]
Brewery waste yeast (*Saccharomyces pastorianus*) immobilized in sodium alginate	Biosorption of Brilliant Red HE-3B reactive dye in batch systems. Depending on the grain size, the following was obtained: q = 222.22 mg/g and R% > 90 (beads wit 2 mm) or q = 151.51 mg/g and R% > 90 for beads with 4 mm.	[29]
Residual *Bacillus* sp. biomass immobilized in sodium alginate	The Thomson and Yoon–Nelson kinetic models can be used to describe to biosorption kinetics of BRed anionic dye onto immobilized residual biomass in a fixed-bed column, when working with flow rates higher than 4.5 mL/min. The maximum biosorption capacity is around 34.742–38.050 mg/g.	[53]
*Saccharomyces pastorianus* residual biomass immobilized in sodium alginate	Biosorption of Orange 16 reactive dye in fluidized-bed column/at lower flow rate (3.8 mL/min) ensures longer contact time, achieving higher capacity (>28 mg/g).	This study

## Data Availability

The original contributions presented in this study are included in the article. Further inquiries can be directed to the corresponding authors.
